# Comparison of tolerability and efficacy of a combination of paracetamol + caffeine and sumatriptan in the treatment of migraine attack: a randomized, double-blind, double-dummy, cross-over study

**DOI:** 10.1007/s10194-012-0484-z

**Published:** 2012-10-02

**Authors:** Luigi Alberto Pini, Simona Guerzoni, Michela Cainazzo, Michela Ciccarese, Maria Pia Prudenzano, Paolo Livrea

**Affiliations:** 1 Headache Study Center, University of Modena & Reggio Emilia, Modena, Italy; 2 Headache Center, “L. Amaducci” Neurological Clinic, University of Bari, Bari, Italy

**Keywords:** Migraine, Acute treatment, Paracetamol–caffeine combination, Sumatriptan

## Abstract

In this study, we compared the efficacy and tolerability of the combination of paracetamol 1,000 mg + caffeine 130 mg (PCF) with sumatriptan 50 mg (SUM) in migraine attacks. This was a multi-center randomized double-blind, double-dummy, cross-over controlled trial. The efficacy was assessed by the sum of pain intensity differences, the curve of mean pain intensity, the number of pain free at 2 h, and the total pain relief. Tolerability was assessed by recording adverse events within 4 h after drug assumption and evaluating the global judgement of patients. The comparison of these parameters did not show differences between the two drugs which resulted absolutely overlapping in pain relief and patients evaluation. In conclusion, we confirm the efficacy and safety of PCF such as SUM in the treatment of migraine attacks.

## Introduction

Migraine is an intermittent neurological disorder, affecting 10–12 % of the western population. In population studies, the prevalence of migraine is approximately 17 % in women and 6 % in men [1]. Migraine is a common, disabling headache disorder, with considerable social and economic impact, and is currently ranked by the World Health Organization as 19th among causes of years lived with disability [2, 3].

Migraine is diagnosed according to the criteria of the International Headache Society (IHS) as a recurrent headache disorder manifesting with attacks lasting 4–72 h. Typical characteristics of the migraine headache are unilateral location, pulsating quality, moderate or severe intensity, aggravation by routine physical activity, and association with nausea and/or photophobia, and phonophobia [4].

Acute migraine attacks can be treated with either unspecific drugs such as acetyl salicylic acid and NSAIDs or specific medicines like triptans and ergot alkaloids. In addition, prokinetic drugs and neuroleptics may be useful [5–7]. Triptans are migraine-specific drugs binding to serotonergic receptors. They are considered first-line therapy for moderate-severe migraine, or mild-moderate attacks unresponsive to nonspecific analgesics [7]. Triptans are more effective in injectable than in oral formulations and should be avoided in patients with a risk for vascular complication [7]. NSAIDs such as ketorolac and naproxen have the advantage of being appropriate for patients with vascular risk factors and they do not cause sedation [7, 8]. NSAIDs are generally well tolerated and may provide benefit even when given late in the migraine attack [8]. However, gastric irritation and occasionally ulceration may complicate treatment with aspirin or other NSAIDs, even when they are used intermittently [7, 8]. The combination of paracetamol and metoclopramide was showed to be superior to paracetamol alone in migraine [9], Paracetamol + caffeine is an useful alternative to NSAID for tension-type headache (TTH); it was compared with placebo in three high-quality studies, showing its superiority over placebo as well as a good tolerability [10, 11].

Till date there is only a large-scale study comparing the early treatment of an association of migraine of sumatriptan 50 mg and a combination of aspirin + acetaminophen + caffeine [12]. This study has been discussed by some authors for some methodological criticisms [13, 14]. Studies comparing paracetamol + caffeine versus sumatriptan at low dose for the treatment of mild-severe migraine treatment are lacking.

The aim of this study was to evaluate the association of paracetamol 1,000 mg + caffeine 130 mg (PCF) to treat acute migraine attacks and compare the efficacy and safety of this product with a gold standard sumatriptan 50 mg by mouth.

## Patients and methods

### Patients

This multi-center study was conducted between May 2011 and April 2012 in two Italian Headache Centers recruiting 108 outpatient volunteers of both genders (one-third male, age 18–62) with a clinical history of episodic migraine fulfilling the following inclusion criteria:

- Diagnosis of migraine fulfilling ICHD-II criteria for migraine with or without aura.
- Mean frequency of 2–8 attacks per month.
- If female, adequate contraception in women of fertile age.
- Daily consumption of at least two cups of coffee.
- Medical history and clinical parameters inconsistent with organic or psychiatric disorders associated with headaches.

Exclusion criteria were:

- Declared hypersensitivity or allergy to paracetamol or sumatriptan.
- Presence of chronic migraine or headache, or medication overuse headache.
- Post-traumatic headache.
- Past or present earth ischemia or myocardial infarction, cerebral ischemic attacks, peripheral vascular diseases, hepatic or renal diseases, mail, severe or uncontrolled hypertension, phenylketonuria, hemolytic anemia.
- Treatment with anticoagulants or antiplatelet drugs.
- Drugs and alcohol abuse, or psychiatric diseases.
- Coagulation disorders, peptic ulcer disease, pancreatic disease, clinically significant renal or hepatic disease, blood hypertension, mild/moderate kidney or liver failure, Gilbert’s syndrome.

The study was conducted, following good clinical practice standards and in accordance with the Declaration of Helsinki (Tokyo version 2004) and stated after independent ethics committee approval for each investigator (Paracaf-emi-010 Code EudraCt 2010-019083-36). Prior to enrolment the patients gave their written informed consent; they were allowed to terminate participation in the trial at any time, without giving reasons. This trial complies with the guidelines for trials of drug treatments in migraine of the IHS [15].

### Study design and treatments

Primary objectives of the study were to show the *efficacy* of the association of paracetamol 1,000 mg and caffeine 130 mg in reducing pain in migraine attacks, and the *tolerability* of this combination in migraine treatment. Secondary objective was to demonstrate the non-inferiority of the PCF association versus SUM in a comparison between these two treatment in migraine attacks.

This was a phase IV study randomized, double-dummy, cross-over, drug-controlled trial. We decided to exclude placebo due to some ethical reasons: this a phase IV study, comparing two well-known active drugs, and our ethical committee did not allow private patients of an active treatment in a study aimed to compare two active drugs.

After obtaining the signature on the informed consent form, the patients were required to treat three subsequent consecutive migraine attacks with the investigational study medications, according to a randomized cross-over sequence computer generated using Microsoft_Access 2003. Each patient was randomly allocated to assume either one PCF and two SUM, or two PCF and one SUM in a randomized sequence treatment. Eligible patients were assigned in sequential order of entry. Access to the randomisation code was strictly controlled and the treatment assignment remained unknown to all parties involved in the trial until database formal lock.

Subjects in all treatment groups received three identical boxes (numbered progressively from 1 to 3, to indicate the exact order in which they should have been used) containing: one soft gel capsule containing one tablet of placebo or sumatriptan 50 mg and one sachet containing paracetamol 1,000 mg + caffeine 130 mg or one sachet containing the excipients and flavor as the active drug.

Blinding was ensured using matched trial supplies, identical in color, size, shape, and taste. At each migraine attack patients would have to take one soft gel capsule and one sachet at the same time. The trial medication was to be taken when the headache occurred, and when the patients would normally have taken their usual analgesic. Other than study medication, patients received rescue medication (usual medication for each patient), to be taken 3 h after the administration of the trial medication, if the pain lasted over the 2 h.

At baseline visit, a complete patients’ medical history and concomitant treatments were recorded, vital signs were measured, and a physical examination was performed by the investigator. Patients were required to record in a headache diary, the date and time of drug ingestion, pain intensity before treatment, pain intensity, pain relief, and adverse events (AEs) after treatment recorded at 1, 2, 3, 4, and 24 h. At the end of 4-h measurement interval or at the time of use of rescue medication, the patients had to record the presence and intensity of AEs. A global judgment of the treatment was also required. The same procedures were repeated for the two subsequent migraine attacks.

### Outcomes

Safety and tolerability were evaluated by comparing vital signs at screening and final visits and by recording AEs. AEs could be recorded by the investigator or by the patient filling in a symptom check-list (including nervousness, palpitation, insomnia, dizziness, abdominal pain, dyspepsia, nausea, vomiting, drowsiness, and fatigue) hourly for 4 h after the study medication ingestion. AEs severity was determined by subjective evaluation of the patient and classified as mild (signs or symptoms easily tolerated), moderate (discomfort sufficient to cause interference with normal activities), and severe (incapacitating with inability to do work or undertake normal activity).

A global assessment of tolerability was expressed by the patient, using a 5-point verbal rating scale (VRS: ‘excellent’, ‘very good’, ‘good’, ‘sufficient’, and ‘poor’).

To assess treatments’ efficacy, intensity of pain (on a 4-point scale: 0 ‘absent’, 1 ‘mild’, 2 ‘moderate’, 3 ‘severe’) and pain relief (on a 5-point scale: 0 ‘no relief’, 1 ‘little relief’, 2 ‘some relief’, 3 ‘much relief’, 4 ‘complete relief’) were evaluated hourly during the 4-h post-dose period.

Based on these two variables, the following parameters were calculated:

- For each patients the sum of pain intensity differences (SPID) was calculated as the sum of differences between pre-dose assessment and every post-dose assessment.
- Total pain relief (TOTPAR), calculated as the sum of every post-dose assessment.

### Statistical analysis

Migraine is a disease with a large inter-individual variability to treatment; to reduce this bias the trial is conducted following a double-blind controlled double-dummy cross-over study versus an active drug.

The study was powered to test the primary hypothesis, namely that paracetamol 1,000 mg + caffeine 130 mg would be non-inferior to SUM as regards the proportion of patients within the 4-h post-dose period.

Assuming a reduction of pain intensity, calculated in a 4-point scale, as recommended by IHS, within 4 h as 1.2 ± 1.3 (SD) in the control study group, we assumed that PCF will be non-inferior when the mean will be Δ = ±0.5. Giving an unilateral α = 0.025 and a 80 % power 110 case for treatment will be enough for statistical analysis.

According to study protocol all the randomized patients who took at least one of the treatments (intention-to-treat, ITT) were evaluated.

Data missing for any scheduled evaluation was replaced by the last observation carried forward (LOCF) procedure. The tolerability endpoints were evaluated using ITT populations; ITT population was employed for efficacy analyses. Descriptive statistics on population was used for demographic and baseline characteristics.

The Fisher exact test (with a 90 % confidence interval) was used to compare the percentage of patients who recorded AEs after each treatment. Besides those recorded by patients in the 4-h post-dose period, all the AEs were classified by the investigator on the basis of treatment received, system involved, severity, and correlation with the investigational medication. The analysis of variance was used to evaluate the differences of vital signs respect to baseline.

The analysis of variance was used to evaluate SPID and TOTPAR. The patient’ preference for one of the treatments was reported as a distribution of frequency. Other statistical tests were used when appropriate.

## Results

108 caucasian patients participated in the study and 92 took at least one of the treatment, whereas 16 patients who filled inclusion criteria did not take any medication. In three cases, the subjects in the following days after randomization explicitly refused to continue the study; In the other 13 cases, when the patients where recalled after 2 months for the second scheduled visit, they refused to continue the protocol for personal reasons. Two patients assumed one treatment and eight subjects assumed two treatments; so the ITT population was of 92 cases and were evaluated for efficacy and tolerability for both the treatments. Globally 264 migraine attacks were evaluated, 131 treated with PCF and 133 treated with SUM. The demographic characteristics and headache history of the patients are summarized in Table 1.

**Table 1:** Demographic data and headache history (ITT, *n* = 92)

Male	31 (33.6 %)			
Female	61 (66.3 %)			
Age (years) (mean ± SD)				
Male	33.6 ± 10.5			
Female	35.6 ± 9.6			
Headache Index	4 ± 3.5			
Usual pain intensity				
Mild	20 (22 %)			
Moderate	49 (53 %)			
Severe	23 (25 %)			
BMI	<18.5	18.5–24.9	25.0–29.9	>30
Male (%)	–	46.2	46.2	7.7
Female (%)	5.9	78.4	11.8	3.9

Headache index = number of day with headache in the observed period

Pain intensity = in a 0–3 scale

With regard to the familial history, 66 (72 %) of cases referred a first-order relative suffering from headache.

### Efficacy

The comparison between the two treatments did not show any difference in pain intensity at baseline both as absolute values and for a Chi square test (*p* = 0.72).

**Fig. 1:**
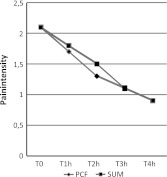
Time course of pain intensity. In both treatment groups since at time *T* *=* 1 h in both groups there was a highly significant difference versus basal time (paired *t* test: *p* < 0.0001)

Figure 1 reports the time course of mean pain intensity for PCF and SUM treatment, while the sum of SPID and the TOTPAR in the *T* 0–4 h period is illustrated in Table 2.

**Table 2:** Sum of pain intensity differences (SPID) and total pain relief (TOTPAR) in the 4-h observation period for the ITT dataset (*n* = 264)

	PCF	SUM	All	*t* test
SPID				
Baseline intensity	2.1 ± 0.7	2.1 ± 0.8	2.1 ± 0.8	
Mean ± SD	3.2 ± 3.8	3.2 ± 3.7	3.2 ± 3.8	*p* = 0.88
TOTPAR				
Mean ± SD	7.0 ± 3.6	7.4 ± 3.6	7.2 ± 3.6	*p* = 0.48

*SD* standard deviation

ANOVA for SPID and TOTPAR showed a positive independent significant variable for intensity of headache at baseline (*p* < 0.001), while no difference between type of treatment (*p* = 0.8849)

Data showed that both the treatments are effective with respect to the baseline, but there were no differences between the treatments.

**Fig. 2:**
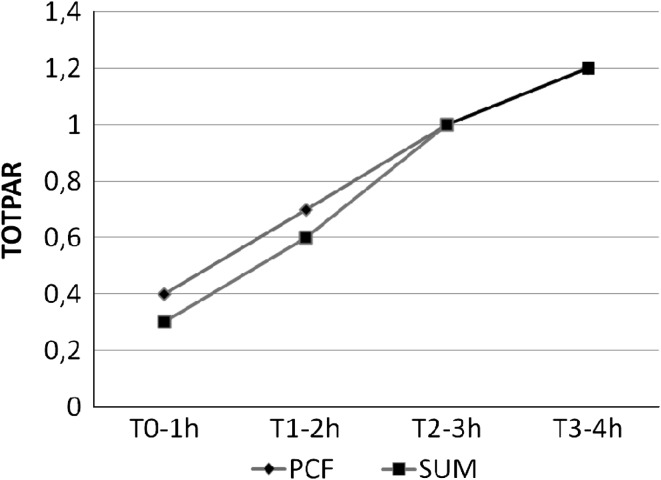
Time course of the total pain relief (TOTPAR) in the ITT dataset. In both treatment groups, since time *T* *=* 0–1 h there was a highly significant difference versus basal time (paired *t* test: *p* < 0.0001)

The time course of TOTPAR is reported in Fig. 2, where similar results are showed. Rescue medication was assumed in 38 % of PCF treatments and 45 % of SUM treatment, without significant differences (Fisher exact test: *p* = 0.3308), even looking at the timing of assumption, i.e., before or 3 h after the first treatment (Fisher exact test: *p* = 0.2245).

### Safety

Taking into account the ITT population and the 264 considered attacks, the number of side effects is reported in Table 3. In about half of recorded attacks, we did not register adverse events (0.9 ± 1.2 and 1.1 ± 1.3 for PCF and SUM, respectively), without differences between the treatments (*t* test: *p* = 0.156). The intensity of side effects was always slight or moderate, all side effects disappeared spontaneously and none requested any modification of scheduled treatment. The only difference between the treatments was a slight increase of referred fatigue in patients assuming SUM.

**Table 3:** Assessment of efficacy and tolerability for each treatment (*n* = 264)

	PCF, *n* (%)	SUM, *n* (%)
Efficacy		
Complete relief	97 (74.1)	96 (72.2)
Much relief	8 (6.5)	9 (6.7)
Some relief	7 (5.3)	8 (6.1)
Little	8 (6.5)	9 (6.7)
No relief	11 (8.4)	11 (8.3)
Tolerability		
Excellent	53 (40.4)	48 (36.4)
Very good	34 (26.2)	33 (24.6)
Good	16 (12.2)	24 (17.8)
Sufficient	17 (12.8)	15 (11.5)
Poor	11 (8.4)	13 (9.7)

Fisher exact test *p* = 0.98 for efficacy and *p* = 0.43 for tolerability

In Table 3 the global assessment of efficacy and tolerability is referred as reported by patients. In Table 4 the number of side effects is reported, and in Table 5 the types of side effects recorded are reported.

**Table 4:** Number of adverse events recorded after drug assumption (dataset = 92 patients and 264 treatments)

Adverse events	Total		Treatments			
			PCF		SUM	
	*N*	%	*N*	%	*N*	%
0 (none)	125	47.3	69	52.7	56	42.1
1	72	27.2	32	24.5	40	30.1
2	40	15.1	18	13.6	22	16.5
3	10	3.9	4	2.7	6	4.5
4	14	5.3	8	6.4	6	4.5
5	2	0.8			2	1.6
6	1	0.4			1	0.7
Total	264	100.0	131	100.0	133	100.0

*t* test: *p* = 0.156, between treatments

**Table 5:** Types of adverse events reported by patients (*N* = 92; 264 treatments)

	Total (*n* = 264)		Treatment				Fisher exact test (*p*)
			PCF (*n* = 131)		SUM (*n* = 133)		
	*N*	%	*N*	%	*N*	%	
None	125	47.3	69	52.7	56	42.1	
Nervousness	32	12.2	13	10.0	19	14.3	0.4125
Palpitazion	27	10.4	12	9.1	15	11.6	0.6607
Insomnia	11	4.1	6	4.5	5	3.6	0.7471
Dizziness	12	4.5	4	2.7	8	6.3	0.3326
Abdominal pain	12	4.5	3	1.8	9	7.1	0.1016
Dyspepsia	21	8.1	11	7.4	12	8.9	0.8067
Nausea	67	25.2	37	28.2	30	22.3	0.3553
Vomito	6	2.3	4	2.7	2	1.8	0.6818
Drowsiness	42	15.8	18	13.6	24	17.9	0.4625
Fatigue	27	10.4	7	5.5	20	15.1	0.0260

## Discussion

Migraine is a widespread condition that in the majority of cases is self-treated by patients, so the use of safe and effective drugs is a reasonable basis for selecting medicines.

Many studies have been conducted to show the efficacy of combination drugs in the treatment of migraine attacks [12, 16, 17].

In a three double-blind, randomized, parallel-group, single-dose, placebo-controlled studies Lipton showed that the combination of acetaminophen, aspirin, and caffeine was highly effective for the treatment of migraine headache pain as well as for alleviating the nausea, photophobia, phonophobia, and functional disability associated with migraine attacks. This drug combination also has an excellent safety profile and is well tolerated [16].

In 2005, Goldstein compared a combination of non-prescription migraine medication (acetaminophen 500 mg, aspirin 500 mg, and caffeine 130 mg) with a prescription migraine product (50 mg sumatriptan) in a randomized, controlled clinical trial in which subjects were treated at the first sign of a migraine attack. He concluded that the combination of acetaminophen, aspirin, and caffeine was significantly more effective (*p* > 0.05) than sumatriptan in the early treatment of migraine, as shown by superiority in summed pain intensity difference, pain relief, pain intensity difference, response, sustained response, relief of associated symptoms, use of rescue medication, disability relief, and global assessments of effectiveness [12].

More recently Diener [17] in a post hoc analysis reported that the fixed combination of ASA (250 mg), paracetamol (200 mg), and caffeine (50 mg) is effective and well tolerated in a broad spectrum from mild-to-severe migraine and TTH severity independent of the headache diagnosis.

Prior confirmed in a double-blind study that acetaminophen 1,000 mg is an effective and well-tolerated treatment for episodic and moderate migraine headache. In addition, acetaminophen generally provided a beneficial effect on associated symptoms of migraine including nausea, photophobia, phonophobia, and functional disability [18].

we some years ago, confirmed the efficacy of the acetaminophen + caffeine association in TTH [11].

In fact, paracetamol exerts its analgesic activity through a direct effect on the central nervous system [19], at least in part mediated by the serotonergic system [20, 21]. Due to its scarce inhibition of peripheral cyclooxygenase: it is better tolerated at gastrointestinal level than NSAIDs, it is only a weak inhibitor of platelets aggregation and does not alter the bleeding time [22].

The association with caffeine is relevant because of the well-known antagonism of adenosine A(2A) and A(2B) receptors, as well as the inhibition of cyclooxygenase activity at some sites, may explain intrinsic antinociceptive and adjuvant actions. When combined with morphine, caffeine can augment, inhibit or have no effect depending on the dose, route of administration, nociceptive test, and species. Low doses of caffeine given systemically inhibit antinociception by several analgesics (acetaminophen, amitriptyline, oxcarbazepine, cizolirtine), probably reflecting block of a component of action involving adenosine A(1) receptors. Clinical studies have demonstrated adjuvant analgesia, as well as some intrinsic analgesia, in the treatment of headache conditions, but not in the treatment of post-operative pain [23], and Diener [24] showed the superiority of the combinations containing caffeine over the association of aspirin and paracetamol alone.

Because the cross-over there were no differences between groups, but it was interesting to note that males showed a BMI significantly superior to the females (Fisher exact test: *p* < 0.001).

Comparing the two treatments, there was a complete overlapping in the efficacy items both for SPID and TOTPAR.

Analyzing the time course of the mean of pain intensity it is interesting to note that at the 24 h almost all patients had resolved their migraine attacks, whereas 41 % of them should assume a rescue medication.

Rescue medication was assumed within both treatments without differences, even when we compared the time of assumption: in fact, there was a small and non-significant difference between SUM treatment where patients assumed in the 38 % of cases the rescue drug at the time 3 versus a 21 % of assumption in PCF treatments (Fisher exact test: *p* = 0.22).

The evaluation of efficacy and tolerability in the patient’s report again showed a complete equivalence of the treatments. In addition, about 80 % of patients for each treatment declared much or complete relief, and a similar percentage referred a very good/excellent tolerability. Even this parameter did not show the differences between the treatments.

We reported the number of total side effects (Table 4) and the more frequent types in Table 5. In this table, there was a significant increase of fatigue in patients assuming SUM compared with cases assuming PCF: This datum result isvery low in the PCF treatment whereas the % in the SUM treatment is similar to the placebo group in our previous study [11].

In conclusion, this trial could have relevant implications for the clinical practice, showing that the simple combination of paracetamol 1,000 mg + caffeine 130 mg seems to be as efficient and safe as sumatriptan 50 mg by mouth, and this could be an important indication for patients suffering from migraine who cannot assume triptans.
